# Supplementary material for: Detailed molecular and epigenetic characterization of the pig IPEC-J2 and chicken SL-29 cell lines
Source: iScience. 2023 Feb 20;26(3):106252. doi: 10.1016/j.isci.2023.106252 (PMC10018572; doi:10.1016/j.isci.2023.106252)
Supplement: Data S1. Complete homer output for identified motifs in Pig IPECJ-2, related to Table 2 — Homer motif analysis results for histone modifications H3K4me1, H3K4me3, H3K27ac, and enhancer elements of pig IPECJ2 cell line. P-values >1e-10 are possible false positives. Within each folder (e.g. peak_files_CTCF) are the html files showing the identified motifs when using homer (e.g. homerResults.html). [file mmc2.zip › S5/Pig_IPECJ_2/peak_files_H3K4me1/homerResults/motif3.similar.html]

motif3

## Information for motif3

G
A
T
C
A
T
C
G
A
G
T
C
A
C
T
G
A
C
G
T
C
G
T
A
T
C
G
A
G
C
T
A
  
Reverse Opposite:  

C
G
A
T
A
G
C
T
G
C
A
T
T
G
C
A
T
G
A
C
C
T
A
G
T
A
G
C
C
T
A
G
  

|  |  |
| --- | --- |
| p-value: | 1e-120 |
| log p-value: | -2.781e+02 |
| Information Content per bp: | 1.409 |
| Number of Target Sequences with motif | 51055.0 |
| Percentage of Target Sequences with motif | 50.85% |
| Number of Background Sequences with motif | 47372.3 |
| Percentage of Background Sequences with motif | 47.16% |
| Average Position of motif in Targets | 149.9 +/- 86.1bp |
| Average Position of motif in Background | 149.9 +/- 87.4bp |
| Strand Bias (log2 ratio + to - strand density) | 0.0 |
| Multiplicity (# of sites on avg that occur together) | 1.50 |
| Motif File: | file (matrix) reverse opposite |

### Similar de novo motifs found

|  |  |  |  |  |  |  |  |
| --- | --- | --- | --- | --- | --- | --- | --- |
| Rank | Match Score | Redundant Motif | P-value | log P-value | % of Targets | % of Background | Motif file |
| 1 | 0.600 | C G T A A C T G A G C T C G T A G T C A C G T A A G T C C G T A | 1e-109 | -251.829367 | 10.75% | 8.71% | motif file (matrix) |
| 2 | 0.680 | G C A T A G C T G A C T T G C A G A T C G A T C C A T G A T C G | 1e-94 | -218.105382 | 60.68% | 57.46% | motif file (matrix) |
| 3 | 0.677 | A C T G A G C T C G T A G T C A T G C A G C A T G C T A G T A C | 1e-88 | -203.675252 | 44.34% | 41.23% | motif file (matrix) |
| 4 | 0.724 | C A G T A C G T G A C T C G T A T G C A T G C A T G A C G C T A T A C G A G T C | 1e-78 | -181.648804 | 32.00% | 29.27% | motif file (matrix) |
